# Cytoreductive Surgery With Hyperthermic Intraperitoneal Chemotherapy: Analysis of Perioperative Risk Factors and Impact on Outcome

**DOI:** 10.7759/cureus.22937

**Published:** 2022-03-07

**Authors:** Joana Paulo, Joana Oliveira, Manuel Silva, Pedro Silva, FIlipe Leite, Rui Valente, Abreu Sousa, Mercês Lobo

**Affiliations:** 1 Anesthesiology, Instituto Português de Oncologia do Porto Francisco Gentil, EPE, Porto, PRT; 2 Epidemiology, Instituto Português de Oncologia do Porto Francisco Gentil, EPE, Porto, PRT; 3 Thoracic Surgery, Instituto Português de Oncologia do Porto Francisco Gentil, EPE, Porto, PRT; 4 General Surgery, Instituto Português de Oncologia do Porto Francisco Gentil, EPE, Porto, PRT

**Keywords:** postoperative management, patient optimization, perioperative outcome, perioperative risk factors, multidisciplinary discussion, cytoreductive surgery and hipec

## Abstract

Background

Cytoreductive surgery plus hyperthermic intraperitoneal chemotherapy (CRS + HIPEC) is an effective treatment option for appropriately selected patients with peritoneal carcinomatosis. Our aim was to analyze a multidisciplinary approach and to study the perioperative risk factors associated with morbidity and mortality.

Methods

We reviewed all patients who underwent CRS + HIPEC from January 2019 till December 2020 at our oncologic center. Patient demographics, risk scores, intraoperative variables, postoperative care, analgesia protocol, and adverse events (AE) within 30 days after treatment were collected and statistically analyzed.

Results

Of the 98 patients evaluated preoperatively by a multidisciplinary team, 39 patients required active optimization. The median age was 61 years, and 67 were women. Most tumors were appendiceal in origin. The median peritoneal cancer index (PCI) score was 12, and the median operative time length (OTL) was 400 minutes. Body mass index, Physiological and Operative Severity Score for the enUmeration of morbidity, PCI score, crystalloid volume, cell concentrates, and OTL were associated with postoperative intensive care unit admission (p <0.05). Epidural analgesia was given to 74 patients. AEs occurred in 39 patients, and 25 of the AEs were classified as mild or moderate. The intraoperative variables associated with development of AEs were anesthesia technique, estimated blood loss, crystalloid volume, cell concentrates, OTL, and analgesia protocol (p <0.05). On multivariate analysis, crystalloid volume >6 L, intravenous sufentanil analgesic protocol, and OTL were associated with 67%, 38%, and 15% increased risk of AE, respectively.

Conclusion

Our study highlighted the importance of a perioperative protocol with a standardized multidisciplinary approach in order to decrease the incidence of postoperative AE.

## Introduction

Peritoneal carcinomatosis used to be considered a palliative and incurable condition with poor survival rates [1] until 1995, when Sugarbaker et al. standardized a surgical approach combined with locoregional chemotherapy, which enabled improvement in quality of life and survival [2]. Over the last decades, cytoreductive surgery (CRS) combined with hyperthermic intraoperative chemotherapy (HIPEC) has evolved as an effective multimodal treatment for selected patients with peritoneal surface malignancies of different origins [3-7]. As any complex procedure, it requires a multidisciplinary and dedicated team and a standard perioperative approach, which includes appropriate patient selection, preoperative optimization, intraoperative protocol, and adequate postoperative care to improve outcomes [8,9]. The creation and management of such meticulous groundwork can only be justified by an increase in quality of care. Therefore, several studies have emphasized the importance of centralizing the procedure in specialized institutions to achieve better outcomes [10-12].

Since 2001, CRS + HIPEC has been performed at our tertiary oncologic hospital, which has a dedicated team with standard protocols in the preoperative, intraoperative, and postoperative periods. In the last five years, a total of 249 procedures were performed, and the surgical and anesthetic approaches have evolved. This study aimed to retrospectively analyze all CRS + HIPEC procedures performed at our center over the last two years, particularly the perioperative risk factors associated with morbidity and mortality.

## Materials and methods

Study design and participants

A retrospective study was performed on all consecutive patients with primary or recurrent peritoneal carcinomatosis who underwent CRS + HIPEC between January 2019 and December 2020 at our hospital. The ethics committee of Instituto Português de Oncologia do Porto Francisco Gentil, EPE issued approval 234/021. Data were retrieved from the patients’ electronic records. No assumptions were made about any missing or unclear information.

Study variables

Data collection included patient demographics, American Society of Anesthesiologists (ASA) classification, preoperative Eastern Cooperative Oncology Group (ECOG) Performance Score, and Physiological and Operative Severity Score for the enUmeration of Mortality and morbidity (P-POSSUM) [13]. Nutritional status was assessed according to body mass index (BMI) and preoperative albumin level. In cases of primary tumor in the gastrointestinal tract, patients were screened by the Malnutrition Universal Screening Tool [14] and were referred for nutritional support if considered high risk (i.e., score of ≥2). In addition, the preoperative hemoglobin level and coagulation profile were reviewed and evaluated for any anomaly based on our laboratory cutoff values. The primary tumor diagnosis was registered.

The intraoperative anesthetic and surgical variables that were recorded and analyzed included anesthesia technique; administered fluids and blood products; estimated blood loss (EBL); hourly urine output; body temperature variation, which was defined as the difference between the highest and lowest temperature measured with nasopharyngeal probe during surgery; and operative time length (OTL), which was defined as the time between induction of and emergence from anesthesia. The amount of infused intraoperative crystalloid fluids was categorized and recorded, as follows: <3 L, 3-6L, and >6 L. Blood product replacement was guided by the EBL and a target hemoglobin level of 8-10 mg/dL. The use of cardiac output (CO) and neuromuscular blockage monitoring were also assessed. The degree of peritoneal disease was evaluated using the peritoneal cancer index (PCI) [15]. The presence of ascites was recorded. Surgical technique was classified as open (i.e., coliseum technique) or closed. Completion of the HIPEC phase within the expected 60-minute duration or interruption before completing the whole cycle was noted.

Postoperatively, admission to the intensive care unit (ICU) or intermediate care unit (IMCU), analgesia protocol, and in-hospital length of stay (LOS) were recorded. The postoperative analgesia protocol was chosen according to the anesthesia technique. Individualized nutritional and physiotherapy support provided in the postoperative period was recorded. Morbidity and mortality were analyzed for the occurrence, severity, and timing of adverse events (AEs), which were stratified according to the Common Terminology Criteria for Adverse Events grading system [16]. Each patient was allocated according to the most severe AE that developed. The development of AEs was evaluated throughout the hospital stay and after discharge within 30 days after surgery, including analysis of patients’ health national records.

Statistical analysis

The patient and clinical characteristics were described using descriptive statistics. Continuous variables were described as median and range, and categorical variables were expressed as frequency (n) and percentage. First, groups were compared using independent samples Wilcoxon-Mann-Whitney test for continuous variables and Chi-square or Fisher’s exact test for categorical variables. Logistic regression analysis was carried out to identify the predictors of AEs within 30 days postoperatively. Univariate models were used to assess the prognostic value of each studied variable. The variables that were significant in the univariate analysis were included in the final multivariate model. Results were considered statistically significant at p value <0.05. All calculations and plots were carried out using R, version 4.1.0 R [17].

## Results

A total of 98 patients underwent CRS + HIPEC during the study period. The demographics, preoperative laboratory parameters and risk scores of the patients are presented in Table 1. The median age was 61 years (range, 32-82 years), and 68.4% were women. The median BMI was 25.6 kg/m2. Appendiceal neoplasm was the most prevalent primary diagnosis (n = 41, 41.8%), and 12 patients had previously undergone CRS + HIPEC.

**Table 1:** Demographic characteristics, preoperative variables, and risk scores ASA: American Society of Anesthesiologists; CRS + HIPEC: cytoreductive surgery with hyperthermic intraoperative chemotherapy; ECOG: Eastern Cooperative Oncology Group; P-POSSUM: Physiological and Operative Severity Score for the enUmeration of Mortality and morbidity

	Total N = 98
Age	
Median (range)	61.0 (32.0–82.0)
Sex	
Female	67 (68.4%)
Male	31 (31.6%)
Body mass index (kg/m^2^)	
Median (range)	25.6 (15.6–43.7)
<18.5	5 (5.1%)
18.5 - 30	72 (73.5%)
>30	21 (21.4%)
Primary diagnosis	
Appendix	41 (41.8%)
Colorectal	27 (27.6%)
Gastric	11 (11.2%)
Mesothelioma	3 (3.1%)
Ovary	16 (16.3%)
Previous CRS + HIPEC	
No	86 (87.8%)
Yes	12 (12.2%)
Hemoglobin (g/dL)	
Median (range)	13.0 (8.8–16.3)
Glomerular filtration rate (mL/min/1.73 m^2^)	
30 - 60	7 (7.1%)
60 - 90	31 (31.6%)
>90	60 (61.2%)
Albumin (g/L)	
<30	4 (4.1%)
30–37	13 (13.3%)
>37	81 (82.7%)
ASA physical status	
II	72 (73.5%)
III	26 (26.5%)
ECOG Performance Status	
0	81 (82.7%)
1	14 (14.3%)
2	3 (3.1%)
P-POSSUM (%)	Median (range)
Morbidity	41.6 (9.6–81.9)
Mortality	8.3 (4.3–78.9)

The median interval between the multidisciplinary oncologic board review of the indications and physiologic conditions for CRS + HIPEC and the procedure was 50 days. All selected patients were evaluated and optimized preoperatively by a multidisciplinary team that involved internal medicine specialists and, when needed, other medical specialists for a median interval of 19.5 days. Of the 39.8% (n = 39) patients who required active management, 16 patients needed additional medical exams, 14 patients needed adjustment of medications, and nine patients required medical assessment by specific specialists. Patients were admitted to the hospital and assessed by the anesthesiologist one day before the procedure.

The median preoperative hemoglobin level was 13.0 g/dL, and 94.9% of the patients (n = 93) had normal preoperative coagulation profile. Preoperative albumin level was >37 g/L in 81 patients. More than 25% of the patients (n = 26) were classified as ASA physical status 3. Most of the patients (82.7%) were classified as ECOG 0. The median morbidity and mortality rates predicted by the P-POSSUM score were 41.6% and 8.3%, respectively.

Data on the intraoperative variables are shown in Table 2. Combined general anesthesia with thoracic epidural analgesia was performed in 74.5% (n = 73). One patient required lumbar epidural catheter placement because of technical difficulties in the thoracic levels. Monitoring was performed according to the standard of care and included pulse oximetry, expired gases, electrocardiogram, invasive blood pressure, hourly urine output, nasopharyngeal temperature, and anesthesia depth through the bispectral index monitor. Neuromuscular monitoring was performed on 43.9% of the patients. The use of central venous line was uncommon.

**Table 2:** Perioperative variables PCEA: patient controlled epidural analgesia; PCI: peritoneal cancer index; ICU: intensive care unit; IMCU: intermediate care unit

		Total N = 98	Median (range)
Anesthesia technique			
Balanced general anesthesia		24 (24.5%)	
Combined general + thoracic epidural		73 (74.5%)	
Combined general + lumbar epidural		1 (1.0%)	
Ascites			
No		84 (85.7%)	
Yes		14 (14.3%)	
Cardiac output monitoring			
No		79 (80.6%)	
Yes		19 (19.4%)	
Estimated blood loss (mL)			200 (50-1500)
Fluid therapy			
Crystalloids			
<3 L		34 (34.7%)	
3–6 L		57 (58.2%)	
>6 L		7 (7.1%)	
Colloids			
Albumin	No	84 (85.7%)	-
	Yes	14 (14.3%)	175 (100-400)
Starch	No	90 (91.8%)	-
	Yes	8 (8.2%)	500 (500-1000)
Gelatin	No	93 (94.9%)	-
	Yes	5 (5.1%)	500 (500-500)
Blood product			
Red cell concentrate	No	85 (86.7%)	
	Yes	13 (13.3%)	600 (300-1200)
Fresh frozen plasma	No	97 (99.0%)	-
	Yes	1 (1.0%)	800 (800-800)
Platelets	No	98 (100%)	-
	Yes	(0%)	-
Urine output (mL/kg/h)			
<0.5		5 (5.1%)	
0.5 - 1		30 (30.6%)	
1 - 2		41 (41.8%)	
>2		22 (22.4%)	
Temperature (°C)			
Minimum			35.5 (34.4–36.4)
Maximum			38.2 (36.7–39.5)
Variation			2.5 (1.3–4.4)
PCI			12.0 (0–39)
Intraperitoneal chemotherapy			
Mitomycin		81 (82.7%)	
Cisplatin		17 (17.3%)	
Operative length (mins)			400 (220-570)
Extubation in the operating room			
No		3 (3.1%)	
Yes		95 (96.9%)	
Analgesia protocol			
PCEA		74 (75.5%)	
Sufentanil		24 (24.5%)	
Postoperative admission			
ICU		33 (33.7%)	
IMCU		65 (66.3%)	
Length of hospital stay (days)			11.0 (5.0–88.0)
Length of ICU stay		33 (33.7%)	1.0 (0.0–5.0)
Length of IMCU stay		65 (66.3%)	2.0 (1.0–8.0)
Physiotherapy evaluation			
No		69 (70.4%)	
Yes		29 (29.6%)	
Nutrition evaluation			
No		63 (64.3%)	
Yes		35 (35.7%)	

Fluid and vasopressor therapy were optimized to maintain blood pressure within 20% of the baseline, and hourly urine output was targeted to achieve >0.5 mL/kg. In 19.4% of the patients, noninvasive CO monitoring (LiDCOrapid®) was used to guide fluid and vasopressor management according to the hemodynamics profile of the patient. The median EBL was 200 mL (range, 50-1,500 mL), and only balanced crystalloid solutions were infused during the intraoperative period, with majority (n = 57, 58.2%) of the patients receiving 3-6 L. A total of 23 patients needed volume resuscitation with colloid fluids; albumin was the most commonly used (n = 14), mainly in cases of preoperative hypoalbuminemia, mucinous ascites, and extensive debulking. Intraoperative blood products were required in 13 patients, and vasopressor support with norepinephrine was needed in six patients. Normothermia was achieved by convective warming blankets and fluid warmers without the need to use active cooling measures. The median variation in temperature was 2.5°C.

**Figure 1:**
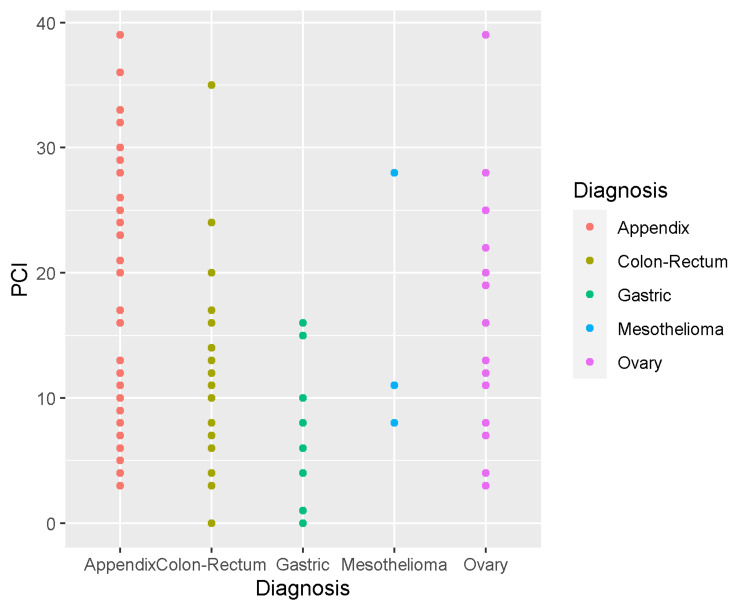
Distribution of peritoneal cancer index according to the primary tumor diagnosis PCI: peritoneal cancer index

The median PCI score was 12 (range, 0-39). As illustrated in Figure 1, the median PCI score was higher in patients with appendiceal and ovarian tumors (16 in both) than in those with colorectal and gastric primary tumors (8.0 and 4.0, respectively).

HIPEC was performed using the open abdomen technique in 62 patients. There were no records of interruptions during the procedure.

In the postoperative period, 33 patients needed ICU admission; only three of these patients were not extubated in the operating room. Table 3 presents the comparison between the ICU and IMCU groups. These groups were similar in terms of demographic characteristics and preoperative risk scores, except for BMI and P-POSSUM morbidity, which were significantly higher in the ICU group (p <0.05). Variation in temperature during the procedure was not related with ICU admission (p >0.05).

**Table 3:** Comparison between ICU and IMCU patients ASA: American Society of Anesthesiologists; ECOG: Eastern Cooperative Oncology Group; ICU: intensive care unit; IMCU: intermediate care unit; PCEA: patient controlled epidural analgesia; PCI: peritoneal cancer index; P-POSSUM: Physiological and Operative Severity Score for the enUmeration of Mortality and morbidity; WMW: Wilcoxon–Mann–Whitney; X2: Chi-square; *statistically significant

	ICU N = 33	IMCU N = 65	p-value	Test
Age (years)				
Median (range)	61.0 (39.0 - 82.0)	60.0 (32.0–80.0)	0.636	WMW
Sex				
Male	12 (36.4%)	19 (29.2%)	0.626	X^2^
Female	21 (63.6%)	46 (70.8%)		
Diagnosis				
Appendix	16 (48.5%)	25 (38.5%)	0.423	Fisher
Colorectal	8 (24.2%)	19 (29.2%)		
Gastric	4 (12.1%)	7 (10.8%)		
Mesothelioma	2 (6.1%)	1 (1.5%)		
Ovary	3 (9.1%)	13 (20.0%)		
Body mass index (kg/m^2^)				
Median (range)	27.6 (17.9–43.7)	25.2 (15.6–39.8)	0.043*	WMW
Hemoglobin (g/dL)				
Median (range)	12.9 (8.9–15.5)	13.0 (8.80–16.3)	0.325	WMW
Albumin (g/L)				
<30	1 (3.0%)	3 (4.6%)	0.601	Fisher
30–37	6 (18.2%)	7 (10.8%)		
>37	26 (78.8%)	55 (84.6%)		
ASA physical status				
II	21 (63.6%)	51 (78.5%)	0.184	X^2^
III	12 (36.4%)	14 (21.5%)		
ECOG Performance Status				
0	24 (72.7%)	57 (87.7%)	0.099	Fisher
1	8 (24.2%)	6 (9.2%)		
2	1 (3.0%)	2 (3.1%)		
P-POSSUM (%)				
Morbidity				
Median (range)	47.0 (27.3 - 81.9)	39.2 (9.6 - 76.0)	0.046*	WMW
Mortality				
Median (range)	9.6 (5.0–28.7)	7.8 (4.3–78.9)	0.147	WMW
Anesthesia technique				
Balanced general anesthesia	26 (78.8%)	47 (72.3%)	0.753	Fisher
Combined general + thoracic epidural	7 (21.2%)	17 (26.2%)		
Combined general + lumbar epidural	0 (0%)	1 (1.5%)		
PCI				
Median (range)	20.0 (0.0–39.0)	8.0 (0.0–39.0)	<0.001*	WMW
Estimated blood loss (mL)				
Median (range)	300.0 (50–1500)	200.0 (50–1200)	0.051	WMW
Crystalloids				
<3 L	5 (15.2%)	29 (44.6%)	0.007*	Fisher
3–6 L	24 (72.7%)	33 (50.8%)		
>6 L	4 (12.1%)	3 (4.6%)		
Red blood cell concentrate (mL)				
No	25 (75.8%)	60 (92.3%)	0.030*	Fisher
Yes	8 (24.2%)	5 (7.7%)		
Temperature (°C)				
Maximum				
Median (range)	38.1 (36.7–39.5)	38.2 (36.9–39.1)	0.865	WMW
Minimum				
Median (range)	35.6 (34.4–36.2)	35.4 (34.5–36.4)	0.197	WMW
Variation				
Median (range)	2.4 (1.4–4.4)	2.6 (1.3–3.8)	0.562	WMW
Operative time length (mins)				
Median (range)	445.0 (340–570)	382.0 (220–515)	<0.001*	WMW
Analgesia protocol				
PCEA	26 (78.8%)	48 (73.8%)	0.773	X^2^
Sufentanil	7 (21.2%)	17 (26.2%)		
Adverse events within 30 days postoperatively				
Yes	17 (51.5%)	22 (33.8%)	0.141	X^2^
No	16 (48.5%)	43 (66.2%)		
Length of hospital stay (days)				
Median (range)	13.0 (7.0–88.0)	10.0 (5.0–51.0)	0.125	WMW

There was a trend of higher EBL in the ICU group than in the IMCU group. Compared with the IMCU group, the ICU group received significantly higher volume of crystalloids (p 0.007), comprised a significantly higher percentage of patients who needed red blood cell (RBC) concentrate transfusion (p 0.03), and had significantly higher PCI score and longer OTL (p <0.001 for both). On the other hand, there were no significant differences in the LOS and AEs that developed within 30 days postoperatively between groups (p >0.05).

Most of the patients (n = 74) received patient controlled epidural analgesia (PCEA) with ropivacaine 0.1% plus sufentanil 0.5 mcg/mL perfusion at a rate of 5-15 mL/hr and combined with intravenous paracetamol 1,000 mg four times a day. Epidural analgesia was maintained for a mean duration of five days. The remaining 24 patients were placed under balanced general anesthesia by infusion of intravenous sufentanil (2.5-20 mcg/hr) plus intravenous paracetamol 1,000 mg four times a day for approximately two days. All patients were assessed by the anesthesiologist every 24 hours until withdrawal of the epidural catheter or suspension of sufentanil infusion; no major complications were identified.

A total of 51 AEs occurred in 39 patients, and 60.2% of our population did not develop any AE. The AEs were classified as grade 1 (mild) in 12 patients, grade 2 (moderate) in 13 patients, grade 3 (severe) in six patients, and grade 4 (life-threatening consequences) in seven patients. One patient died on the 14th postoperative day because of multiorgan failure, which was classified as grade 5 AE.

Table 4 shows all the AEs recorded throughout in-hospital stay.

**Table 4:** Adverse events during in-hospital stay ICU: intensive care unit; IMCU: intermediate care unit; PONV: postoperative nausea and vomiting; UTI: urinary tract infection

		Early postoperative period		Ward stay
		ICU	IMCU	
Neurologic (ischemic stroke)		-	1	-
Pulmonary (atelectasis, pleural effusion, pneumonia)		-	-	5
Cardiovascular (dysrhythmias, hemodynamic instability, hypertension)		4	-	3
Renal (acute renal failure, percutaneous nephrostomy)		1	-	1
Gastrointestinal (ileus)		-	-	1
Hematologic (anemia, thrombocytopenia)		1	4	2
Infectious (surgical site infections, UTI, febrile neutropenia, bacteremia)		-	1	4
Others				
PONV		1	2	-
Epidural catheter adverse events (malfunction, exteriorization)		1	2	-
Iatrogenic nerve lesion (femoral nerve)		1	-	-
ICU readmission			-	3
Fistula / dehiscence / abscess	Conservative treatment	-	-	7
	Surgical intervention	-	-	5
Death		-	-	1

Cardiovascular and hematologic complications were the most frequent AEs in the ICU and IMCU, respectively. Notably, one patient developed ischemic stroke with brachial hemiplegia during IMCU stay. For ward stay, fistula, dehiscence, or abscess was the most frequent AE that developed. Seven patients were treated by conservative approach, including antibiotic therapy and interventional radiology procedures, but five of these patients required surgical intervention. Pulmonary AEs, including atelectasis, pleural effusion, and pneumonia, were detected in five patients. Postoperative nausea and vomiting (PONV), which led to delay in oral refeeding, was reported in only three patients. There were three cases of epidural catheter malfunction or exteriorization that required changes in the analgesic protocol. Three patients needed ICU readmission because of cardiovascular AE and septic shock.

On the 30th postoperative day, 10 of 98 patients remained hospitalized because of AEs. On univariate linear regression analysis, occurrence of AE during the postoperative period was associated with a mean increase of 15.2 days in LOS (p <0.001). After hospital discharge and until the 30th day postoperatively, 10 patients returned to the hospital because of AE; five (two cases of ileus and three cases of surgical site infection) of them were treated on an outpatient basis, whereas the other five patients needed readmission because of fistula, dehiscence, or abscess. One of these readmissions needed surgical intervention.

Table 5 presents the perioperative factors associated with AE development within 30 days postoperatively.

**Table 5:** Perioperative variables associated with the development of adverse events ASA: American Society of Anesthesiologists; ECOG: Eastern Cooperative Oncology Group; ICU: intensive care unit; IMCU: intermediate care unit; PCEA: patient controlled epidural analgesia; PCI: peritoneal cancer index; P-POSSUM: Physiological and Operative Severity Score for the enUmeration of Mortality and morbidity; WMW: Wilcoxon–Mann–Whitney; X2: Chi-square; *statistically significant

	Adverse events		p-value	Test
	NO (N = 59)	YES (N = 39)		
Age (years)				
Median (range)	60.0 (32.0–80.0)	62.0 (33.0–82.0)	0.632	WMW
Sex				
Male	17 (28.8%)	14 (35.9%)	0.606	X^2^
Female	42 (71.2%)	25 (64.1%)		
Diagnosis				
Appendix	21 (35.6%)	20 (51.3%)	0.190	Fisher
Colorectal	18 (30.5%)	9 (23.1%)		
Gastric	5 (8.5%)	6 (15.4%)		
Mesothelioma	2 (3.4%)	1 (2.6%)		
Ovary	13 (22.0%)	3 (7.7%)		
Body mass index (kg/m^2^)				
Median (range)	25.5 (15.6–38.2)	25.7 (18.9–43.7)	0.535	WMW
Hemoglobin (g/dL)				
Median (range)	13.4 (9.4–16.3)	12.7 (8.8–15.7)	0.172	WMW
Albumin (g/L)				
<30	1 (1.7%)	3 (7.7%)	0.291	Fisher
30–37	7 (11.9%)	6 (15.4%)		
>37	51 (86.4%)	30 (76.9%)		
ASA physical status				
II	48 (81.4%)	24 (61.5%)	0.052	X^2^
III	11 (18.6%)	15 (38.5%)		
ECOG Performance Status				
0	49 (83.1%)	32 (82.1%)	0.665	Fisher
1	9 (15.3%)	5 (12.8%)		
2	1 (1.7%)	2 (5.1%)		
P-POSSUM (%)				
Morbidity				
Median (range)	37.9 (9.6-76.0)	45.5 (27.3-81.9)	0.059	WMW
Mortality				
Median (range)	7.6 (4.3-78.9)	9.1 (5.0-28.7)	0.202	WMW
Anesthesia technique				
Balanced general anesthesia	9 (15.3%)	15 (38.5%)	0.015*	Fisher
Combined general + thoracic epidural	49 (83.1%)	24 (61.5%)		
Combined general + lumbar epidural	1 (1.7%)	0 (0%)		
PCI				
Median (range)	11.0 (0.0–39.0)	14.0 (0.0–36.0)	0.132	WMW
Estimated blood loss (mL)				
Median (range)	150.0 (50-700)	300.0 (100–1500)	<0.001*	WMW
Crystalloids				
<3 L	27 (45.8%)	7 (17.9%)	<0.001*	Fisher
3–6 L	32 (54.2%)	25 (64.1%)		
>6 L	0 (0%)	7 (17.9%)		
Red cell concentrate (mL)				
No	56 (94.9%)	29 (74.4%)	0.008*	X^2^
Yes	3 (5.1%)	10 (25.6%)		
Temperature (°C)				
Maximum				
Median (range)	38.1 (36.7–39.2)	38.2 (36.9–39.5)	0.708	WMW
Minimum				
Median (range)	35.4 (34.4–36.4)	35.6 (34.8–36.2)	0.168	WMW
Variation				
Median (range)	2.7 (1.3–3.8)	2.4 (1.4–4.4)	0.433	WMW
Operative time length (minutes)				
Median (range)	370.0 (220–520)	440.0 (340–570)	<0.001*	WMW
Analgesia protocol				
PCEA	50 (84.7%)	24 (61.5%)	0.018*	X^2^
Sufentanil	9 (15.3%)	15 (38.5%)		
Postoperative admission				
ICU	16 (27.1%)	17 (43.6%)	0.141	X^2^
IMCU	43 (72.9%)	22 (56.4%)		

Patient demographics, preoperative laboratory parameters, and risk scores were not associated with AE development. Although not significant, there was a trend of higher ASA and P-POSSUM morbidity scores in the group that developed AEs than in the group that did not develop AEs. On the other hand, intraoperative variables, such as EBL, volume of crystalloids infused, RBC concentrate transfusion, OTL, anesthesia technique, and analgesia protocol, were significantly associated with AE development (p <0.05). On multivariate logistic regression analysis, mean OTL, intravenous analgesic protocol with sufentanil, and crystalloid volume >6 L were the only significant factor that increased the risk of development of AE within 30 days postoperatively (p <0.05) by 15%, 38% and 67%, respectively.

## Discussion

In this study, we presented data on the perioperative management and risk factors associated with morbidity and mortality in patients who underwent CRS + HIPEC for primary or recurrent peritoneal carcinomatosis. To our best knowledge, this study was the largest research carried out in a single center over a short period of time.

Preoperative optimization

Our outcomes relied on patient selection after review by a multidisciplinary oncologic board discussion. Moreover, evaluation of all patients by a multidisciplinary team enabled a standardization and effective preoperative optimization for a median interval of 19.5 days between assessment and surgery. This strategy aimed to improve patient outcomes through evaluation by other medical specialties. Low molecular weight heparin prophylaxis before surgery and compliance with fasting guidelines were also implemented.

Intraoperative protocol

The surgical routine during the cytoreductive phase was based on inspection and PCI determination, proceeding from primary tumor resection to total peritonectomy and removal of all macroscopic peritoneal disease. General anesthesia combined with thoracic epidural analgesia enabled an opioid sparing technique in the majority of patients. Intermittent pneumatic compression devices, balanced crystalloid fluid therapy, normothermia maintenance, restrictive blood transfusion policy, and early extubation may have also contributed to our results on patient outcomes.

Operative time length

Other authors have reported OTLs between 360 and 625 minutes [8,18]. The median OTL in this study population was 400 mins and was significantly longer in the group with AEs than in the group without AEs (440 mins vs. 370 mins, p <0.001). For each extra hour of OTL, the risk for AE development was increased by 15%, as also reported in other studies [19,20].

Fluid therapy

We advocate the use of balanced crystalloid solution instead of 0.9% saline because of its association with relatively low incidence of hyperchloremic acidosis and, therefore, kidney dysfunction and mortality [21]. Similar to previous reports [22,23], our study showed a significantly larger volume of crystalloid given to patients who developed AEs than in those who did not develop AEs. Human albumin was selectively used in cases of previous hypoalbuminemia, extensive debulking, and mucinous ascites.

In terms of hemodynamic monitoring, the randomized trial of Luca Colantonio et al. [24] on goal-directed versus standard fluid therapy in CRS + HIPEC procedures concluded that the use of a goal-directed fluid therapy improved the outcome in terms of the incidence of major abdominal and systemic postoperative complications and LOS. Although less than 20% of our patients underwent CO monitoring, there was no significant difference in outcomes between patients on goal-directed therapy and the ones who received standard fluid therapy, as likewise reported by other studies [25]. Although CO monitoring was not found to be relevant in our population, we believed that if it was more widely used, targeted fluid therapy could standardize the care given to patients at our institution and further improve our outcomes.

Epidural

A classical xifopubic midline incision with extensive multivisceral resections to achieve a complete cytoreduction has been associated with intense pain, both acute and chronic. Despite the potential risks associated with epidural analgesia, which includes hematoma secondary to coagulopathy and infectious complications secondary to immunosuppression, several reports in literature underscored not only the benefits on pain control, postoperative pulmonary complications, and recovery of bowel function but also the guaranteed safety in CRS + HIPEC procedures [26-28].

Indeed, the most recent enhanced recovery after surgery (ERAS) guidelines [29] strongly recommended the routine use of thoracic epidural analgesia in CRS + HIPEC procedures to achieve pain relief, spare opioids, and hasten the resumption of bowel function. In our population, >75% of the patients had adequate pain relief with epidural analgesia and no major neuraxial complications reported. Furthermore, the group with epidural analgesia had a significantly reduced incidence of postoperative AE development than in the group that received intravenous sufentanil.

Postoperative nausea and vomiting

CRS + HIPEC procedure is considered a highly emetogenic surgery. In our practice, we use the Apfel score [30] to determine the need to administer intravenous dexamethasone 4 mg upon anesthetic induction and intravenous ondansetron 4 mg at the end of surgery, with additional intravenous droperidol 0.625-1.25 mg for patients with previous history of PONV. In addition, the postoperative analgesic protocols included antiemetics. The fact that we had only three cases of delayed oral intake because of PONV highlighted the efficacy of our approach. Moreover, the low opioid doses with epidural analgesia further contributed to the low PONV incidence.

Early extubation

According to the ERAS guidelines [29], almost all patients (96.9%) who underwent CRS + HIPEC were extubated in the operating room. As reported in other studies, we believed that the use of epidural catheter contributed to these results, because there was a reduced opioid requirement in both the intraoperative and postoperative periods. Moreover, a relatively early ambulation can lead to early recovery and reduced incidence of postoperative AE [28,29].

ICU/IMCU

Our analysis showed that not all patients who have undergone CRS + HIPEC needed ICU admission. We believed that our patients were correctly assigned to each destination, given that the AE incidence was not significantly different between ICU and IMCU patients, despite the higher risk in the preoperative risk assessment and intraoperative findings that can predispose to worse outcomes in the former. These results may be explained by the proportionate increase in the postoperative care that these patients required and the ICU staff experience. Preoperative evaluation using performance and physical status scores were not the best predictors of patient allocation, given that neither ASA classification or ECOG score was significantly different between groups; only the BMI and P-POSSUM morbidity scores were significantly different between groups. Intraoperative variables, including OTL, surgical complexity evaluated by PCI, crystalloid volume, and RBC concentrate transfusion were the most significant for predicting ICU admission.

Ward stay

Early mobilization and spirometer use was encouraged throughout the postoperative period in all patients. Additionally, one-third of our population (high-risk patients and cases of long LOS) was evaluated in order to establish an individualized physiotherapy plan. Likewise, specific evaluation by the nutrition team was carried out in 35% of the patients who were identified in the preoperative screening as having or being at risk of malnutrition and in cases of long LOS.

Limitations

The main limitations of our study included its retrospective design. Although the fact that the involvement of a single institution with a dedicated team increased the internal validity of our study, extrapolation of our results to all centers may not be applicable.

## Conclusions

In summary, our center had a relevant number of patients who underwent CRS + HIPEC with a perioperative protocol that included several established recommendations in the 2020 guidelines. This approach included preoperative patient selection and optimization by a multidisciplinary team, standardized surgical and anesthetic practice, and allocation of patients into an appropriate care unit in the postoperative period based on the preoperative and intraoperative assessment. In our population, the use of epidural analgesia, compared with intravenous sufentanil, prevented postoperative AE development, and each extra hour of OTL increased the risk of AE development. Moreover, intraoperative infusion of >6 L of crystalloid volume was associated with an increased risk of AE development. The ERAS guidelines highlighted the lack of strong evidence and reinforced the need to investigate and publish better scientific evidence. Therefore, we believed that sharing our experience may contribute to the daily practice in this field of surgery.
